# Electrospun Nanofibers Made of Silver Nanoparticles, Cellulose Nanocrystals, and Polyacrylonitrile as Substrates for Surface-Enhanced Raman Scattering

**DOI:** 10.3390/ma10010068

**Published:** 2017-01-14

**Authors:** Suxia Ren, Lili Dong, Xiuqiang Zhang, Tingzhou Lei, Franz Ehrenhauser, Kunlin Song, Meichun Li, Xiuxuan Sun, Qinglin Wu

**Affiliations:** 1 Key Biomass Energy Laboratory of Henan Province, Zhengzhou 450008, Henan, China; rensuxia2004@163.com (S.R.); dongli2050@163.com (L.D.); zhangxiuqiang@126.com (X.Z.); 2 Audubon Sugar Institute, Louisiana State University Ag Center, St. Gabriel, LA 70776, USA; FEhrenhauser@agcenter.lsu.edu; 3 School of Renewable Natural Resources, Louisiana State University Ag Center, Baton Rouge, LA 70803, USA; ksong2@lsu.edu (K.S.); MLi@agcenter.lsu.edu (M.L.); xsun17@lsu.edu (X.S.)

**Keywords:** surface-enhanced Raman scattering, electrospinning, cellulose nanocrystal, polyacrylonitrile, silver nanoparticles

## Abstract

Nanofibers with excellent activities in surface-enhanced Raman scattering (SERS) were developed through electrospinning precursor suspensions consisting of polyacrylonitrile (PAN), silver nanoparticles (AgNPs), silicon nanoparticles (SiNPs), and cellulose nanocrystals (CNCs). Rheology of the precursor suspensions, and morphology, thermal properties, chemical structures, and SERS sensitivity of the nanofibers were investigated. The electrospun nanofibers showed uniform diameters with a smooth surface. Hydrofluoric (HF) acid treatment of the PAN/CNC/Ag composite nanofibers (defined as p-PAN/CNC/Ag) led to rougher fiber surfaces with certain pores and increased mean fiber diameters. X-ray diffraction (XRD) and X-ray photoelectron spectroscopy (XPS) results confirmed the existence of AgNPs that were formed during heat and HF acid treatment processes. In addition, thermal stability of the electrospun nanofibers increased due to the incorporation of CNCs and AgNPs. The p-PAN/CNC/Ag nanofibers were used as a SERS substrate to detect p-aminothiophenol (p-ATP) probe molecule. The results show that this substrate exhibited high sensitivity for the p-ATP probe detection.

## 1. Introduction

Surface-enhanced Raman scattering (SERS) is a hypersensitive, noninvasive, and powerful analytical technique for the ultimate identification of small molecules through enhancing Raman signals of the molecules located in the region of noble metals or their nanostructures [1,2]. Noble-metal nanoparticles (e.g., Au and Ag) have become increasingly important because of their unique features of localized surface Plasmon resonance, resulting in excellent activity and good sensitivity as SERS substrates [1,3,4,5]. However, using Au nanoparticles (AuNPs) or AgNPs colloids directly is often inconvenient in real-world applications due to the difficulties in handling fluids. To reduce or eliminate this problem, a feasible method is to fabricate flexible nanostructured SERS substrates. Electrospinning is a facile and efficient way to prepare SERS active substrates with well-dispersed AgNPs in polymer nanofibers [5,6,7]. 

Electrospinning has become a practical technique for production of nanofibers with diameters commonly ranging from tens to hundreds of nanometers because of its versatility and cost-effective setup [8,9,10]. These nanofibers usually have sufficient surface area and high porosity features, enabling them to be assembled and processed for a variety of applications [7], including biomimetic processes [11], sensors [10,12], and environmental fields [13]. Recently, there have been some studies on the manufacture of electrospun nanofibers containing metal nanoparticles, in which SERS activities of the prepared nanofibers were explored [1,3,7,8,14]. Compared to the common SERS substrates, such as porous silicon modified with silver nanoparticles [2], electrospun nanofiber substrates have several merits such as large specific surface area, high porosity, good mechanical properties, suitability for surface modification, and easy accessibility [7]. Hence, it is of significance to develop flexible electrospun nanofibers as SERS substrates to enhance detection sensitivity. As such, there is a great interest in the development of new methods for the fabrication of functional and multicomponent electrospun nanofibers loaded with highly active metallic nanoparticles. Recently, polymer-metal nanocomposites have received a great attention owing to their excellent physical and chemical properties. Among the polymers, Polyacrylonitrile (PAN) is a polymer that has been intensively studied due to its high dielectric constant desirable for electrospinning [15]. PAN can be used as a carrier to disperse other materials to obtain electrospun multi-phase nanostructures [16]. 

Nanocellulose is a sustainable nanomaterial, well-known for its abundance, biodegradability, and biocompatibility [17]. Nanocellulose can be used as a substrate for loading a range of different nanomaterials [18,19] and the resultant nanocomposites have merits from both of the individual nanomaterials. To date, SERS of AuNPs and AgNPs has been extensively studied [3,4,20,21]. The performance of AuNPs/nanocellulose or AgNPs/nanocellulose nanocomposites as SERS substrates has also been reported in several studies [22,23,24,25,26,27,28,29]. For example, Limei Tian et al. [22] described a gravity-assisted filtration method for biosynthesized bacterial nanocellulose based SERS substrate fabrication. Chook et al. [26] studied the preparation of highly porous cellulose nanofiber-AgNPs nanocomposite through an environmentally benign method, which was demonstrated for its SERs activity and catalytic properties. Among the nanocellulose materials, cellulose nanocrystals (CNCs) draw special attention due to excellent mechanical properties, high purity, simple surface chemistry, and cost-efficient production. However, to the best of our knowledge, there are no studies exploring the potential use of CNCs for SERS substrate fabrication. The introduction of CNCs might provide a new route to combine the performance of hydrophilic CNC phase and hydrophobic PAN phase. The potential hydrogen bonding interaction between the hydroxyl group of CNCs and the cyano groups of PAN may present a good foundation for fabricating metallic salts/CNC/polymer nanostructures. 

Low-dimensional silicon (Si) nanostructures have been intensely investigated and the electrochemistry of Si has spurred intense research activity in microelectronic technology [30]. Silicon exhibits reduced electrochemical properties in hydrofluoric acid (HF) solutions, allowing the electrodeless reduction of metal ions to metallic particles. 

Herein, we describe a facile synthesis approach to functionalize PAN with CNCs and AgNPs and produce electrospun nanostructures for SERS substrate applications. In the system, SiNPs were also used as a template for electrodeless deposition of Ag. The p-aminothiophenol (p-ATP) was selected as the model material for SERs detection because it is one of the most typical and excellent materials in SERS measurement. 

## 2. Materials and Methods

### 2.1. Materials

Polyacrylonitrile (PAN, average M_w_ = 150,000 g/mol), *N*,*N*-dimethylformamide (DMF), Hydrofluoric acid (HF), and silver nitrate (AgNO_3_) were purchased from Sigma-Aldrich (St. Louis, MO, USA). Cellulose nanocrystals (CNCs, 7.4% w/w solid content, aqueous suspension) were purchased from Blue Goose Biorefineries Inc. (Edmonton, AB, Canada). Silicon nanoparticles (Si NPs) were purchased from Shanghai St-nano Science and Technology Co., Ltd. (Shanghai, China). The p-aminothiophenol (p-ATP) was purchased from Shanghai Mackin Biochemical Co. Ltd. (Shanghai, China). All chemicals were used directly without further treatment.

### 2.2. Preparation

#### 2.2.1. Fabrication of Electrospun Nanofibers 

PAN powder was added in DMF and the mixture was stirred for 24 h at room temperature to make a solution with a PAN concentration of 10% (w/v, PAN/DMF). Dried CNC powder was added in DMF and the mixture was vigorously stirred for 48 h at room temperature to make a suspension with a CNC concentration of 0.2% (w/v, CNC/DMF). Then the obtained CNC suspension was added into the PAN solution under magnetic stirring for 2 h to get the homogeneous PAN-CNC-DMF suspension. SiNPs and AgNO_3_ were added into the above suspension with magnetic stirring for 10 h to produce the final electrospinning suspension with 0.15 wt % and 1.5 wt % concentrations for SiNPs and AgNO_3_, respectively. The suspension was kept in the dark to avoid the decomposition of AgNO_3_. Afterward, the above suspensions were heated to 90 °C in a water bath for 15 min to reduce part of the Ag ions to AgNPs through a reaction: $2{\mathrm{AgNO}}_{3}=2\mathrm{Ag}+2{\mathrm{NO}}_{2}+O_{2}$. In this process, AgNO_3_ played the role not only of a reductant, but also of an oxidant. The suspensions containing CNCs, PAN, SiNPs, and AgNPs were cooled to room temperature for electrospinning. The compositions of the electrospinning precursor suspensions are shown in Table 1.

Each prepared precursor suspension was loaded into a 5 mL Becton-Dickinson (BD) plastic syringe (Franklin Lakes, NJ, USA) and equipped with a stainless-steel needle tip (internal diameter 0.584 mm). Then the syringe was driven by an electric syringe pump (Chemyx Fusion 100, Stafford, TX, USA) set at a fixed flow rate of 0.5 mL/h. The needle was connected to a high-voltage power supply (Gamma High Voltage Research, Ormond Beach, FL, USA), and the positive DC voltage was fixed at 18 kV. An aluminum foil-covered metal plate was horizontally placed to collect the nanofiber with a needle tip-to-plate distance of 18 cm. The collected nanofiber mats were dried in a vacuum oven at 40 °C to remove the residual solvent. The obtained electrospun nanomats are designated as PAN, PAN/Ag, PAN/CNC/Ag, and PAN/CNC/Ag/Si.

The PAN/CNC/Ag/Si nanofibers were further immersed into a HF solution (5 wt %) for 30 min to remove SiNPs embedded in the nanofibers. The process also led to electrodeless conversion of AgNPs from Ag ions. The nanomats were then washed with deionized water for several times and dried in a vacuum oven at 40 °C. The HF acid treated PAN/CNC/Ag/Si nanofiber mats contained fibers with some pores and are designated as p-PAN/CNC/Ag.

#### 2.2.2. Characterization

A rheometer (AR2000ex, TA Instruments, New Castle, DE, USA) was used to measure the shear viscosities of the precursor suspensions with a 40-mm cone-plate geometry. The viscosity for each suspension was recorded at shear rates ranging from 0.1 to 1000 s^−1^ at 25 °C. In order to avoid solvent evaporation, a solvent trap cover was used during the measurements.

Surface morphologies of the electrospun nanomats were observed using a FE-SEM (FEI Quanta™ 3D FEG Dual Beam SEM/FIB, Hillsboro, OR, USA) under an accelerating voltage of 5 kV. The surfaces of the mats were coated with a thin layer of gold before observation. The diameters of the nanofibers were obtained using the image processing software (ImageJ 1.48) through measuring 50 randomly chosen individual nanofibers from the FE-SEM images. To characterize the dispersion and size of Ag nanoparticles in the electrospun fibers, high resolution transmission electron microscopy (HRTEM, FEI Talos F200S, Hillsboro, OR, USA) operating at an accelerating voltage of 200 kV was used.

The crystalline structures of the electrospun nanostructures were determined using Bruker/Siemens D5000 X-ray diffractometer (XRD-Siemens Co., Wittelsbacherplatz, Munich, Germany). The experiments were conducted from 5° to 80° using a scan speed of 0.2°/min with Cu-K_α_ radiation (λ = 1.54 Å, Voltage = 45 kV, and I = 40 mA).

X-ray photoelectron spectroscopy (XPS) analysis was performed on a Specs PHOIBOS-100 spectrometer (SPECS, Berlin, Germany) with an Al-K_α_ irradiation (1486.61 eV) at 10 kV and 10 mA current. Survey spectra were recorded from 0 to 1200 eV with a pass energy of 40 eV and a step size of 1.0 eV.

An FTIR spectrometer (Alpha, Bruker Optics Inc., Billerica, MA, USA) was used to observe the chemical structure of the electrospun nanostructures. The resolution of the IR spectrometer was 4 cm^−1^ and each sample was scanned in the range of 4000–400 cm^−**1**^.

Thermal properties of the samples were studied with a SDT Q600 analyzer (TA Instruments, New Castle, DE, USA). The spontaneous thermogravimetric (TG), differential thermogravimetric (DTG), and differential scanning calorimetry (DSC) data of the samples were recorded from room temperature to 800 °C at a heating rate of 10 °C/min. The test was conducted in N_2_ atmosphere (flow rate = 100 mL/min).

For the SERS tests, p-ATP was used as the probe molecule. A sample of 10 mg from each prepared nanostructure material was immersed in 1.0 × 10^−4^ mol/L of p-ATP solution for 30 min. Then, the material was dried at 60 °C and attached to silicon substrates for measurement. The SERS spectra of each nanostructure containing p-ATP probe molecules were acquired at a randomly selected spot on the substrates using a LabRAM HR800 confocal Raman spectroscopy (Horiba Jobin Yvon, Bensheim, Germany) from 400 to 1800 cm^−1^. An yttrium aluminum garnet (YAG) laser 532 nm was used as excitation source.

## 3. Results and Discussion

### 3.1. Properties of Electrospinning Suspension

**Figure 1:**
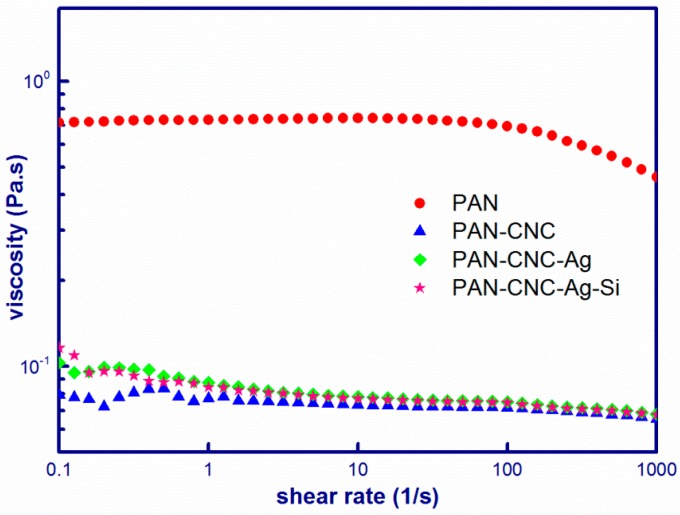
Shear viscosity-shear rate relationships for PAN, PAN/CNC, PAN/CNC/Ag, and PAN/CNC/Ag/Si.

The most important factor for successful preparation of electrospun nanostructures is to form a homogeneous precursor suspension. In this study, homogeneous suspensions consisting of PAN, CNCs, AgNPs, and SiNPs were obtained using DMF as solvent. The shear viscosities of the precursor suspensions at the range of 0.1–1000 s^−1^ are shown in Figure 1.

The PAN solution showed the Newtonian behavior at low shear rates. At higher shear rates, the PAN solution viscosity decreased with increase in the shear rate. During this process, the PAN molecular chains were gradually disentangled by increased shear stress, and thus a pseudoplastic behavior of the solution was observed at higher shear rates. For PAN/CNC suspension, the plateau disappeared and the viscosity displayed nearly Newtonian behavior within the whole investigated shear region. The viscosity of PAN/CNC suspension was much lower compared to that of PAN, which might be caused by the distortion of PAN molecular chain-chain interactions with the CNCs. Compared to PAN/CNC, the PAN/CNC/Ag and PAN/CNC/Ag/Si suspensions showed almost the same viscosities except that these two suspensions had slightly higher viscosity at low shear rates, which was caused by the increased concentrations of the electrospinning suspensions after the addition of Ag NPs and Si NPs.

### 3.2. Morphologies of Electrospun Nanofibers

**Figure 2:**
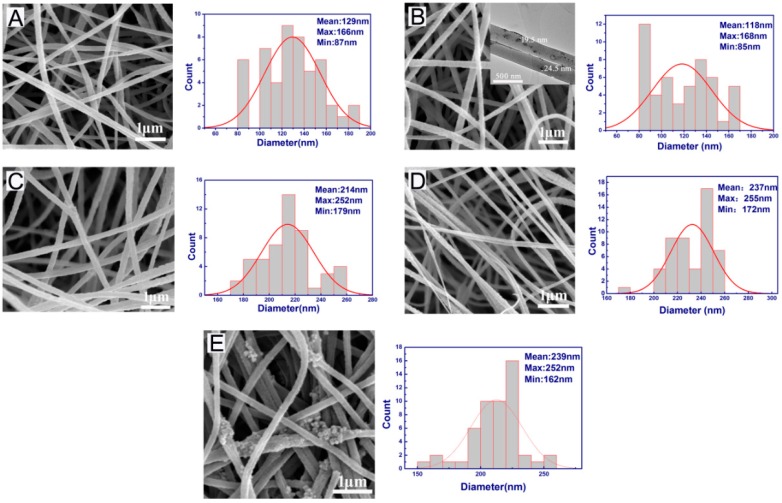
FE-SEM micrographs and corresponding fiber diameter distribution of electrospun nanofibers. (**A**) PAN; (**B**) PAN/Ag; (**C**) PAN/CNC/Ag; (**D**) PAN/CNC/Ag/Si; and (**E**) p-PAN/CNC/Ag. Inset in (**B**) is the TEM micrograph of PAN/Ag showing Ag particles.

Figure 2 shows the SEM pictures of pure PAN, PAN/Ag, PAN/CNC/Ag, PAN/CNC/Ag/Si, and p-PAN/CNC/Ag fiber mats. It can be seen that all the spun nanofibers exhibited smooth surfaces (except Figure 2E), uniform diameters. The nanofiber mats had highly porous structures. For p-PAN/CNC/Ag, many AgNPs were found on the surfaces of nanofibers from the electrodeless deposition of Ag on silicon for the HF/AgNO_3_ system [30]. The reaction can be outlined by the two half-cell reactions. First, anodic reactions (Equations (1) and (2)) occurred through Si atoms oxidization and then the electrons were supplied for the Ag^+^ reduction (cathode reaction, Equation (3)).

(1) $$\mathrm{Si}\left(s\right)+2H_{2}O\to {\mathrm{SiO}}_{2}+4H^{+}+4e^{-}$$

(2) $${\mathrm{SiO}}_{2}+6\mathrm{HF}\to H_{2}{\mathrm{SiF}}_{6}+2H_{2}O$$

(3) $${\mathrm{Ag}}^{+}+e^{-}\to {\mathrm{Ag}}^{0}\left(s\right)$$

The corresponding size distributions of the above nanofibers are listed in Figure 2 (next to each SEM picture). The average diameter of pure PAN, PAN/Ag, PAN/CNC/Ag, and PAN/CNC/Ag/Si were 129 ± 14, 118 ± 14, 214 ± 12, and 237 ± 11 nm, respectively. The average diameter of PAN/Ag nanofibers was lower compared to that of PAN nanofibers because of the increased electrical conductivity of PAN/Ag suspensions, which led to the increase of the surface charge of the polymer jet. The polymer jet in the electrospinning process experienced stronger elongation forces, resulting in a thinner diameter of the nanostructure [31]. The inset in Figure 2B is the high-resolution TEM micrograph of the PAN/Ag fibers. As shown, Ag NPs dispersed well in the electrospun nanofibers and had diameters about 20 nm. The increasing tendency of the average diameters of PAN/CNC/Ag, and PAN/CNC/Ag/Si nanofibers (compared with PAN/Ag system) might be caused by the addition of inorganic particles (AgNPs and SiNPs) in the precursor suspensions [32]. The introduction of inorganic particles led to lower viscosity of the electrospun suspension, resulting in larger diameter electrospun nanofibers. For the p-PAN/CNC/Ag electrospun nanostructure, no obvious change of the diameter was observed because the HF acid treatment process did not change the macroscopic structure of the electrospun nanofibers.

### 3.3. Crystalline Structure 

**Figure 3:**
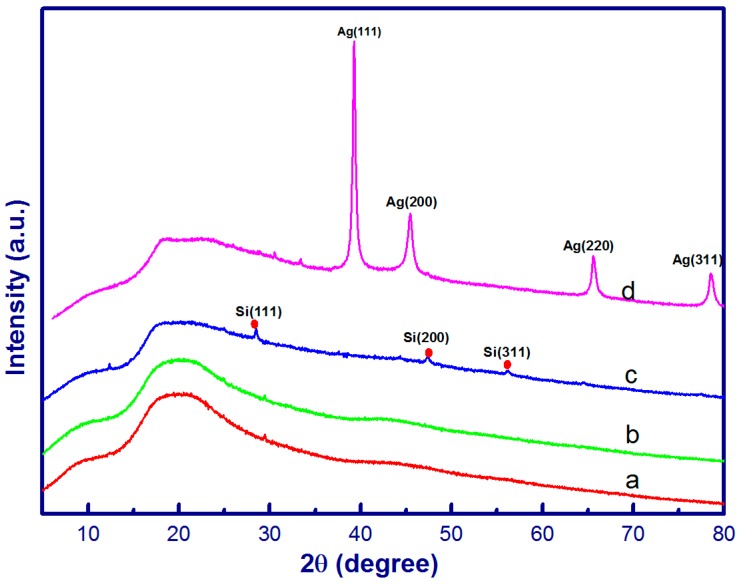
XRD patterns of PAN (**a**); PAN/CNC/Ag (**b**); PAN/CNC/Ag/Si (**c**); and p-PAN/CNC/Ag (**d**).

The crystalline structure of electrospun nanostructures was verified by XRD as shown in Figure 3. For PAN and PAN/CNC/Ag nanofibers, a broad peak centered at 2θ = 20° is indicative of the amorphous nature of PAN. While there was no obvious diffraction peaks that can be assigned to AgNPs for both PAN/CNC/Ag and PAN/CNC/Ag/Si nanofibers, XPS results proved the presence of AgNPs (Supplementary Materials Figures S1 and S2). CNC diffraction peaks were also not found in these electrospun nanofibers. The contents of actual AgNPs after the heat treatment and CNCs in these electrospun nanofibers were most likely too low to be detected using the XRD technique. For both PAN/CNC/Ag and PAN/CNC/Ag/Si nanomats, the concentration of Ag ions in the electrospun precursor suspension was 30 wt % based on the weight of PAN weight (10%). During the 15-min heat treatment process, only a small number of Ag ions were converted to solid AgNPs and their content was below the detection limit of the XRD technique [33]. The PAN/CNC/Ag/Si nanostructures exhibited three diffraction peaks with 2θ values at around 28.5°, 47.4°, and 56.2°, corresponding to the (111), (200), and (311) crystal planes of SiNPs, respectively [34]. Apparently, XRD methods detected Si in the fiber more effectively at 3% silicon loading level in the fiber. After HF acid treatment, a typical XRD pattern of p-PAN/CNC/Ag nanofibers showed diffraction peaks with 2θ values located at around 38.2°, 44.2°, 64.4°, and 77.4°, which, respectively, corresponded to the (111), (200), (220), and (311) crystallographic planes of the face-centered cubic structure of AgNPs [3,35]. This result indicates that additional AgNPs were formed during the HF acid treatment process, which allowed XRD technique to successfully detect them and the results are consistent with the SEM results.

### 3.4. Chemical Composition and Structure of p-PAN/CNC/Ag Nanofibers

**Figure 4:**
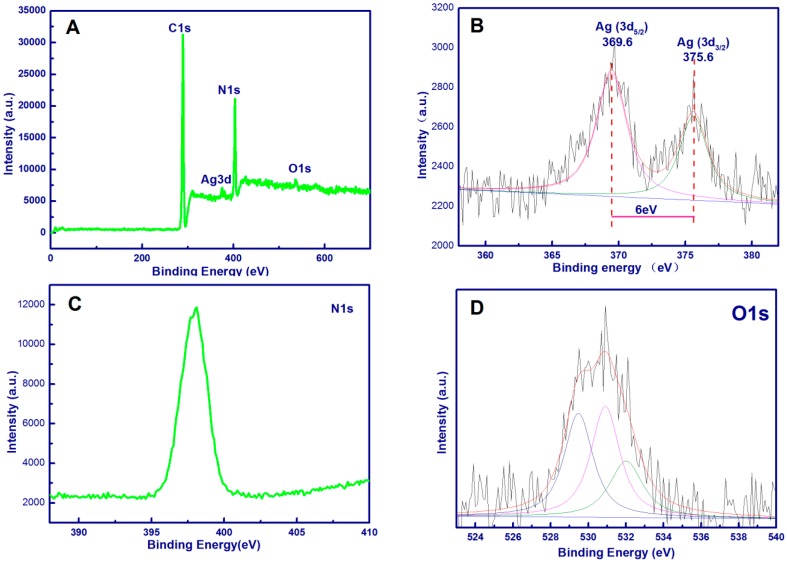
XPS full scan spectra of P-PAN/CNC/Ag nanofibers (**A**); the XPS spectra of Ag 3d (**B**); XPS spectra of N 1s (**C**); and XPS spectra of O 1s (**D**).

XPS measurement was used to analyze the chemical composition and electronic structures of the prepared p-PAN/CNC/Ag nanofibers (Figure 4). As shown in Figure 4A, peaks for C, N, O, and Ag were found in the p-PAN/CNC/Ag nanofibers. The high-resolution XPS of Ag 3d is shown in Figure 4B, p-PAN/CNC/Ag nanomats exhibited two specific peaks with binding energies of 369.6 and 375.6 eV, corresponding to Ag 3d_5/2_ and Ag 3d_3/2_ energy levels, respectively. The spine energy separation is 6 eV, reflecting the metallic nature of Ag [1,3,35]. The N 1s levels (Figure 4C) exhibited only one peak at 398.4 eV, which is assigned to nitrogen atoms present in nitrile structures (CN) [36]. Figure 4D is the high resolution XPS of O1s, the peak of –OH in the nanomat located at 532.0 eV. It shifted to lower values from literature reported values because of the decreased electron cloud density of O in –OH of the p-PAN/CNC/Ag system, suggesting that O chelated with silver ions [1]. It suggested that Ag ions were chelated between the hydroxyl sites of CNCs and Ag ions, not with the cyano groups of PAN. CNCs in these electrospun nanostructures acted as a bridge between PAN by H-bonds and the metallic nanostructure, laying a good foundation for preparing metallic/CNC/PAN nano composites. The other two peaks located at 530.9 eV and 529.5 eV were assigned to C=O and Al_2_O_3_, which may be caused by the residual solvent and aluminum foil used to collect the fibers [1]. 

**Figure 5:**
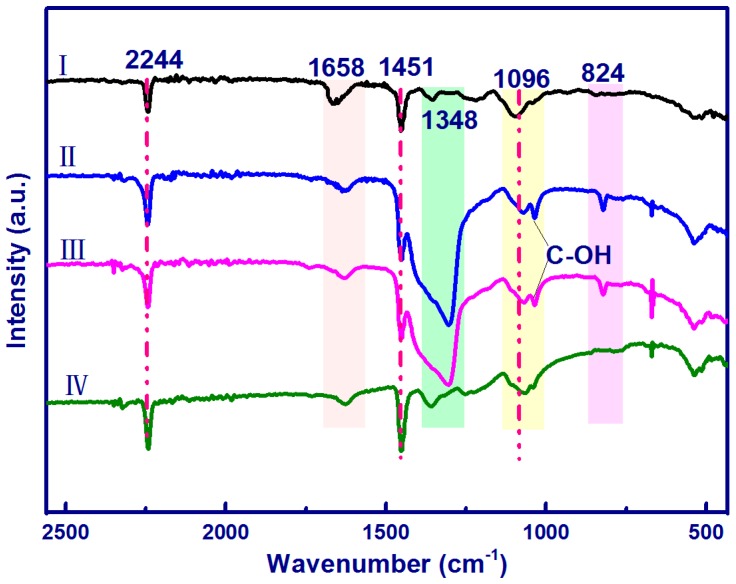
FTIR spectra of the electrospun nanofibers: (**I**) PAN; (**II**) PAN/CNC/Ag; (**III**) PAN/CNC/Ag/Si; and (**IV**) p-PAN/CNC/Ag/Si.

The FTIR spectra of the electrospun nanostructures are shown in Figure 5. For pure PAN nanofibers, distinctive peaks at 2244, 1451, and 1096 cm^−1^ corresponded to CN stretching vibration, CH_2_ bending vibration, CH wagging, and skeletal vibration of the PAN molecular chain, respectively [32,37,38]. The peaks around 1658 cm^−1^ are attributed to the O–H bending of adsorbed water. The peaks at 1340–1380 are assigned to the aliphatic CH group vibrations of CH_2_. Compared to those of pure PAN, the spectra of PAN/CNC/Ag and PAN/CNC/Ag/Si nanofibers show new peaks at 1034 cm^−1^ and 824 cm^−1^ corresponding to the characteristic absorption bands O–H of CNCs and C–H rock respectively [31], which indicates the CNC existence in the electrospun nanofibers. There was no observable –CN– bond vibration shift, suggesting that there were no chemical bonds or interactions between –CN– groups in the PAN and AgNPs. After HF acid treatment, more AgNPs were formed on the surface of the nanofibers. 

### 3.5. Thermal Properties

**Figure 6:**
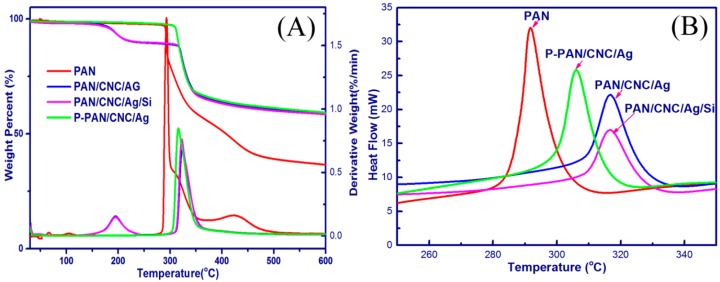
TG and DTG curves (**A**); and DSC curves (**B**) of the electrospun nanofibers.

The TGA, TGC, and DSC curves of the electrospun nanostructures are shown in Figure 6. For the PAN nanofibers, there was no obvious weight loss before 288 °C. The temperature range between 288 and 450 °C was the main decomposition stage, due to the disconnection of polymer chains [38]. For the PAN/CNC/Ag and PAN/CNC/Ag/Si nanofibers, there were two decomposition stages. The first stage was between 167 °C and 220 °C, which may be caused by the decomposition of CNCs in the nanofibers. The onset temperature of these two samples was 167 °C, which was lower than that of pure PAN (288 °C). After the HF treatment, the onset temperature of the p-PAN/CNC/Ag material was 306 °C, which was higher than that of PAN/CNC/Ag and PAN/CNC/Ag/Si materials. It may be due to the formation of AgNPs during the electrodeless deposition process, which served as a protection layer and increased the material’s thermal stability. This was consistent with the DTG peaks of the as-spun nanofibers, which were also shifted toward higher temperatures.

The DSC curves of PAN nanofibers showed a sharp exothermic peak at 288 °C (Figure 6B), which was caused by the cyclization reactions of nitrile groups in PAN [32,39]. The PAN polymer chains can convert to a heteroaromatic ladder structure through the cyclization process [32]. After the addition of CNCs to Ag and Si, the electrospun nanostructures for PAN/CNC/Ag and PAN/CNC/Ag/Si both exhibited a wider peak and had cyclization reactions at higher temperatures (316 °C) than that of the pure PAN (288 °C). This phenomenon may be caused by the changes of PAN cyclization mechanism from a radical mechanism to a slower ionic mechanism, typical for PAN copolymers [39]. The CNCs, Ag, and Si added to PAN and PAN/CNC slowed down the cyclization reactions of PAN, avoiding the fusion of the electrospun nanofibers during the thermal process. For the p-PAN/CNC/Ag samples, the cyclization reactions occurred at a lower temperature (306 °C) than these of the PAN/CNC/Ag and PAN/CNC/Ag/Si systems. It might be caused by the structural changes of the polymers during the HF treatment process. 

### 3.6. SERS Activity of p-PAN/CNC/Ag Nanofilm as Substrates

**Figure 7:**
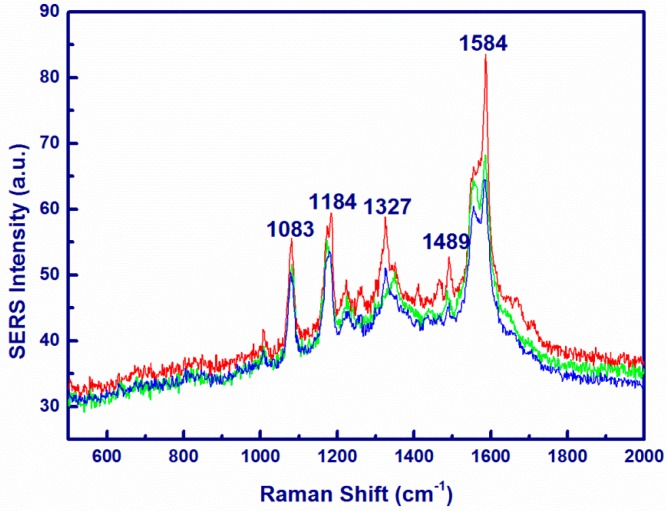
SERS spectrum of p-ATP (1 × 10^−4^ M) recorded on randomly selected three spots on the surface of p-PAN/CNC/Ag.

The PAN/Ag nanofibers substrates without CNCs showed no SERS for p-ATP probe molecule (Figure S3). The PAN/CNC/Ag nanofibers substrates showed only poor SERS for the p-ATP probe molecule (Figure S4). However, when the synthesized p-PAN/CNC/Ag system was used as an SERS substrate, a remarkable enhancement was observed. The Raman spectra of p-PAN/CNC/Ag nanomats with p-ATP as a probe molecule are shown in Figure 7. According to literature [1], the main Raman peaks of solid p-ATP are located at 1091 cm^−1^ (C–S stretching mode ) and 1598 cm^−1^ (C–C ring breathing) [1]. In the recorded SERS spectra, ν (C–S) at 1083 cm^−1^, δ (C–H) at 1184 cm^−1^, ν (C–C) and δ (C–H) at 1327 cm^−1^, δ (C–H) and ν (C–C) at 1499 cm^−1^, and ν (C–C) at 1584 cm^−1^ were significantly enhanced. It should be pointed out that the enhancement degree varied with different band width. Thus, there is no single enhancement factor over the entire spectra. Since the band at 1583 cm^−1^ can demonstrate the band for b2 bending modes [1], the band at the 1583 cm^−1^ position was selected for estimating the SERS enhancement factor (EF) values. The normal Raman spectrum of solid p-ATP is shown in Figure S5. According to the reported method [1], the EF value for the current system was estimated to be 3.9 × 10^3^. These results demonstrate that p-ATP adsorbed to the surface of the p-PAN/CNC/Ag nanomats and resulted in the remarkable Raman enhancement. The CNCs in the PAN/CNC/Ag system acted as a coordination agent for the formation of AgNPs. In short, the as-spun nanostructures can be used as effective SERS substrates for cost-effective SERS applications. The PAN matrix allows the nanofibers to keep their morphology after immersing into sample solution, a distinct advantage when compared with published data in other work [40].

## 4. Conclusions

The p-PAN/CNC/Ag composite nanofibers were successfully fabricated by a convenient electrospinning technique using PAN, AgNPs, SiNPs, and CNCs as main fiber composition. The fibers exhibited excellent activities for SERS. Silver nitrate was reduced to AgNPs during the heat and the HF acid treatment processes, which was confirmed by XRD and XPS results. The electrospun nanofibers showed uniform diameters with a smooth surface, but for the p-PAN/CNC/Ag composite nanofibers the surface became rougher with pores and the fiber diameter increased. In addition, the incorporation of CNCs, AgNPs, and HF treatment increased the thermal stability of the electrospun nanofibers. Ultimately, the novel material p-PAN/CNC/Ag composite nanofibers were successfully demonstrated as a useful SERS substrate for applying to the p-ATP probe molecules. The functionalized nanofibers pave a new way in developing a membrane for sensors, catalytic materials, and other devices.

## Figures and Tables

**Table 1:** Compositions of different electrospinning suspensions *.

Sample	C_PAN_ (wt %)	C_CNC_ (wt %)	C_Ag_ (wt %)	C_Si_ (wt %)
PAN	10	0	0	0
PAN/Ag	10	0	30	0
PAN/CNC/Ag	10	2	30	0
PAN/CNC/Ag/Si	10	2	30	3

* C_PAN_ is the concentration of PAN in the suspensions. C_CNC_, C_Ag_, and C_Si_ is the content based on the weight of PAN.

## Supplementary Materials

The following are available online at www.mdpi.com/1996-1944/10/1/68/s1. Figure S1, The XPS spectra of Ag 3d for PAN/CNC/Ag. Figure S2, the XPS spectra of Ag 3d for PAN/CNC/Ag/Si. Figure S3, SERS spectrum of p-ATP (1 × 10^−4^ M) recorded on randomly selected spots on the surface of PAN/Ag. Figure S4, SERS spectrum of p-ATP (1 × 10^−4^ M) recorded on randomly selected spots on the surface of PAN/CNC/Ag.

## References

[1] Yang T., Yang H., Zhen S.J., Huang C.Z. (2015). Hydrogen-Bond-Mediated in Situ Fabrication of AgNPs/Agar/PAN Electrospun Nanofibers as Reproducible SERS Substrates. ACS Appl. Mater. Interfaces.

[2] Harraz F.A., Ismail A.A., Bouzid H., Al-Sayari S.A., Al-Hajry A., Al-Assiri M.S. (2015). Surface-enhanced Raman scattering (SERS)-active substrates from silver plated-porous silicon for detection of crystal violet. Appl. Surf. Sci..

[3] Mondal S., Rana U., Malik S. (2015). Facile Decoration of Polyaniline Fiber with Ag Nanoparticles for Recyclable SERS Substrate. ACS Appl. Mater. Interfaces.

[4] Su X., Wang Y., Wang W., Sun K., Chen L. (2016). Phospholipid Encapsulated AuNR@Ag/Au Nanosphere SERS Tags with Environmental Stimulus Responsive Signal Property. ACS Appl. Mater. Interfaces.

[5] Amarjargal A., Tijing L.D., Kim C.S. (2015). Simple fabrication of Ag nanoparticle-impregnated electrospun nanofibres as SERS substrates. Bull. Mater. Sci..

[6] He D., Hu B., Yao Q.-F., Wang K., Yu S.-H. (2009). Large-Scale Synthesis of Flexible Free-Standing SERS Substrates with High Sensitivity: Electrospun PVA Nanofibers Embedded with Controlled Alignment of Silver Nanoparticles. ACS Nano.

[7] Zhang L., Gong X., Bao Y., Zhao Y., Xi M., Jiang C., Fong H. (2012). Electrospun Nanofibrous Membranes Surface-Decorated with Silver Nanoparticles as Flexible and Active/Sensitive Substrates for Surface-Enhanced Raman Scattering. Langmuir.

[8] Celebioglu A., Aytac Z., Umu O.C.O., Dana A., Tekinay T., Uyar T. (2014). One-step synthesis of size-tunable Ag nanoparticles incorporated in electrospun PVA/cyclodextrin nanofibers. Carbohydr. Polym..

[9] Shafiei M., El-chami I., Rintoul L., Bahreyni B. (2017). Morphology of electrospun poly(ethylene oxide) ultra-fine fibres with incorporated MoO_3_ nanoparticles. Mater. Des..

[10] Khoshaman A.H., Li P.C.H., Merbouh N., Bahreyni B. (2012). Highly sensitive supra-molecular thin films for gravimetric detection of methane. Sens. Actuators B Chem..

[11] Lin J., Wang X., Ding B., Yu J., Sun G., Wang M. (2012). Biomimicry via Electrospinning. Crit. Rev. Solid State Mater. Sci..

[12] Wang H., Wang D., Peng Z., Tang W., Li N., Liu F. (2013). Assembly of DNA-functionalized gold nanoparticles on electrospun nanofibers as a fluorescent sensor for nucleic acids. Chem. Commun..

[13] Thavasi V., Singh G., Ramakrishna S. (2008). Electrospun nanofibers in energy and environmental applications. Energy Environ. Sci..

[14] Li F., Kang W., Cheng B., Dong Y. (2015). Preparation and catalytic behavior of hollow Ag/carbon nanofibers. Catal. Commun..

[15] Ji L., Zhang X. (2008). Ultrafine polyacrylonitrile/silica composite fibers via electrospinning. Mater. Lett..

[16] Ji L., Yao Y., Toprakci O., Lin Z., Liang Y., Shi Q., Medford A.J., Millns C.R., Zhang X. (2010). Fabrication of carbon nanofiber-driven electrodes from electrospun polyacrylonitrile/polypyrrole bicomponents for high-performance rechargeable lithium-ion batteries. J. Power Sources.

[17] Wei H., Rodriguez K., Renneckar S., Vikesland P.J. (2014). Environmental science and engineering applications of nanocellulose-based nanocomposites. Environ. Sci. Nano.

[18] Sehaqui H., Liu A., Zhou Q., Berglund L.A. (2010). Fast Preparation Procedure for Large, Flat Cellulose and Cellulose/Inorganic Nanopaper Structures. Biomacromolecules.

[19] Cai J., Liu S., Feng J., Kimura S., Wada M., Kuga S., Zhang L. (2012). Cellulose–Silica Nanocomposite Aerogels by In Situ Formation of Silica in Cellulose Gel. Angew. Chem. Int. Ed. Engl..

[20] Wu Y., Li P., Yang B., Tang X. (2016). Designing and fabricating composites of PNIPAM@Au nanorods with tunable plasmon coupling for highly sensitive SERS detection. Mater. Res. Bull..

[21] Huang Y., Miao Y.-E., Ji S., Tjiu W.W., Liu T. (2014). Electrospun Carbon Nanofibers Decorated with Ag–Pt Bimetallic Nanoparticles for Selective Detection of Dopamine. ACS Appl. Mater. Interfaces.

[22] Tian L., Jiang Q., Liu K.-K., Luan J., Naik R.R., Singamaneni S. (2016). Bacterial Nanocellulose-Based Flexible Surface Enhanced Raman Scattering Substrate. Adv. Mater. Interfaces.

[23] Zhang L., Li X., Ong L., Tabor R.F., Bowen B.A., Fernando A.I., Nilghaz A., Garnier G., Gras S.L., Wang X. (2015). Cellulose nanofibre textured SERS substrate. Colloids Surf. A Physicochem. Eng. Asp..

[24] Wei H., Vikesland P.J. (2015). pH-Triggered Molecular Alignment for Reproducible SERS Detection via an AuNP/Nanocellulose Platform. Sci. Rep..

[25] Wei H., Rodriguez K., Renneckar S., Leng W., Peter J. (2015). Preparation and evaluation of nanocellulose-gold nanoparticle nanocomposites for SERS applications. Analyst.

[26] Chook S.W., Chia C.H., Chan C.H., Chin S.X., Zakaria S., Sajab M.S., Huang N.M. (2015). A porous aerogel nanocomposite of silver nanoparticles-functionalized cellulose nanofibrils for SERS detection and catalytic degradation of rhodamine B. RSC Adv..

[27] Park M., Chang H., Jeong D.H., Hyun J. (2013). Spatial deformation of nanocellulose hydrogel enhances SERS. BioChip J..

[28] Marques P.A., Nogueira H.I., Pinto R.J., Neto C.P., Trindade T. (2008). Silver-bacterial cellulosic sponges as active SERS substrates. J. Raman Spectrosc..

[29] Pinto R.J., Marques P.A., Martins M.A., Neto C.P., Trindade T. (2007). Electrostatic assembly and growth of gold nanoparticles in cellulosic fibres. J. Colloid Interface Sci..

[30] Peng K.Q., Hu J.J., Yan Y.J., Wu Y., Fang H., Xu Y., Lee S.T., Zhu J. (2006). Fabrication of Single-Crystalline Silicon Nanowires by Scratching a Silicon Surface with Catalytic Metal Particles. Adv. Funct. Mater..

[31] Huang S., Zhou L., Li M.C., Wu Q., Kojima Y., Zhou D. (2016). Preparation and Properties of Electrospun Poly (Vinyl Pyrrolidone)/Cellulose Nanocrystal/Silver Nanoparticle Composite Fibers. Materials.

[32] Song K., Wu Q., Zhang Z., Ren S., Lei T., Negulescu I.I., Zhang Q. (2015). Porous Carbon Nanofibers from Electrospun Biomass Tar/Polyacrylonitrile/Silver Hybrids as Antimicrobial Materials. ACS Appl. Mater. Interfaces.

[33] Niño-Martínez N., Martínez-Castañón G.A., Aragón-Piña A., Martínez-Gutierrez F., Martínez-Mendoza J.R., Facundo R. (2008). Characterization of silver nanoparticles synthesized on titanium dioxide fine particles. Nanotechnology.

[34] Liu W.-R., Guo Z.-Z., Young W.-S., Shieh D.-T., Wu H.-C., Yang M.-H., Wu N.-L. (2005). Effect of electrode structure on performance of Si anode in Li-ion batteries: Si particle size and conductive additive. J. Power Sources.

[35] Chen K., Shen Z., Luo J., Wang X., Sun R. (2015). Quaternized chitosan/silver nanoparticles composite as a SERS substrate for detecting tricyclazole and Sudan I. Appl. Surf. Sci..

[36] Pirlot C., Mekhalif Z., Fonseca A., Nagy J.B., Demortier G., Delhalle J. (2003). Surface modifications of carbon nanotube/polyacrylonitrile composite films by proton beams. Chem. Phys. Lett..

[37] Chang H., Chien A.-T., Liu H.C., Wang P.-H., Newcomb B.A., Kumar S. (2015). Gel Spinning of Polyacrylonitrile/Cellulose Nanocrystal Composite Fibers. ACS Biomater. Sci. Eng..

[38] Wang W., Li W., Gao C., Tian W., Sun B., Yu D. (2015). A novel preparation of silver-plated polyacrylonitrile fibers functionalized with antibacterial and electromagnetic shielding properties. Appl. Surf. Sci..

[39] Frank E., Steudle L.M., Ingildeev D., Spörl J.M., Buchmeiser M.R. (2014). Carbon Fibers: Precursor Systems, Processing, Structure, and Properties. Angew. Chem. Int. Ed..

[40] Yang H., Huang C.Z. (2014). Polymethacrylic acid-facilitated nanofiber matrix loading Ag nanoparticles for SERS measurements. RSC Adv..

