# Supplementary material for: Electrospun Nanofibers Made of Silver Nanoparticles, Cellulose Nanocrystals, and Polyacrylonitrile as Substrates for Surface-Enhanced Raman Scattering
Source: Materials (Basel). 2017 Jan 14;10(1):68. doi: 10.3390/ma10010068 (PMC5344577; doi:10.3390/ma10010068)
Supplement: Supplementary file 1 [file materials-10-00068-s001.pdf]

Supplementary Materials: Electrospun Nanofibers Made of Silver Nanoparticles, Cellulose Nanocrystals and Polyacrylonitrile as Substrates for Surface-Enhanced Raman Scattering

Suxia Ren, Lili Dong, Xiuqiang Zhang, Tingzhou Lei, Franz Ehrenhauser, Kunlin Song, Mei-Chun Li, Xiuxuan Sun and Qinglin Wu

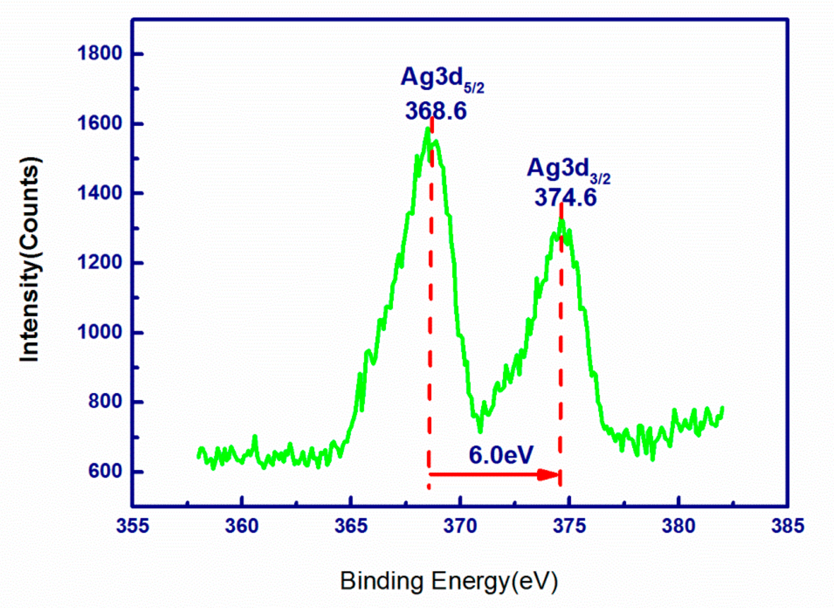

Figure S1. The XPS spectra of Ag 3d for PAN/CNC/Ag.

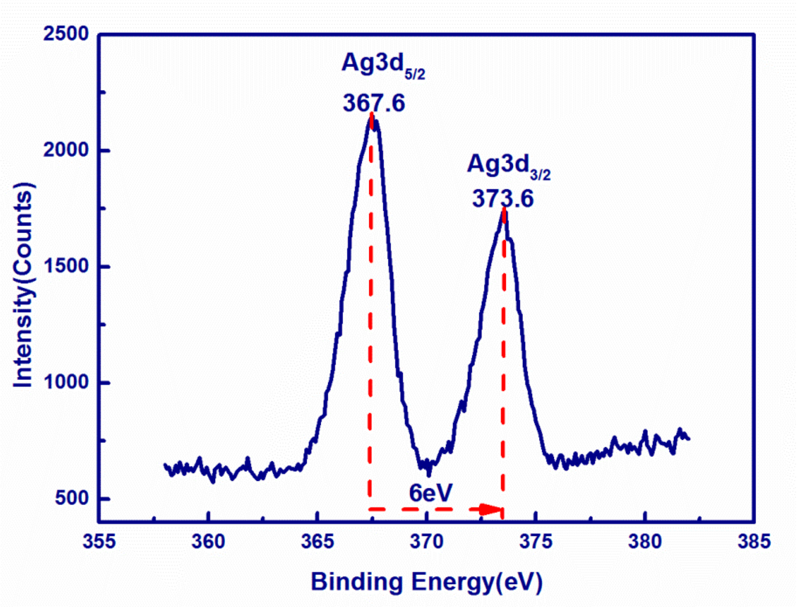

Figure S2. The XPS spectra of Ag 3d for PAN/CNC/Ag/Si.

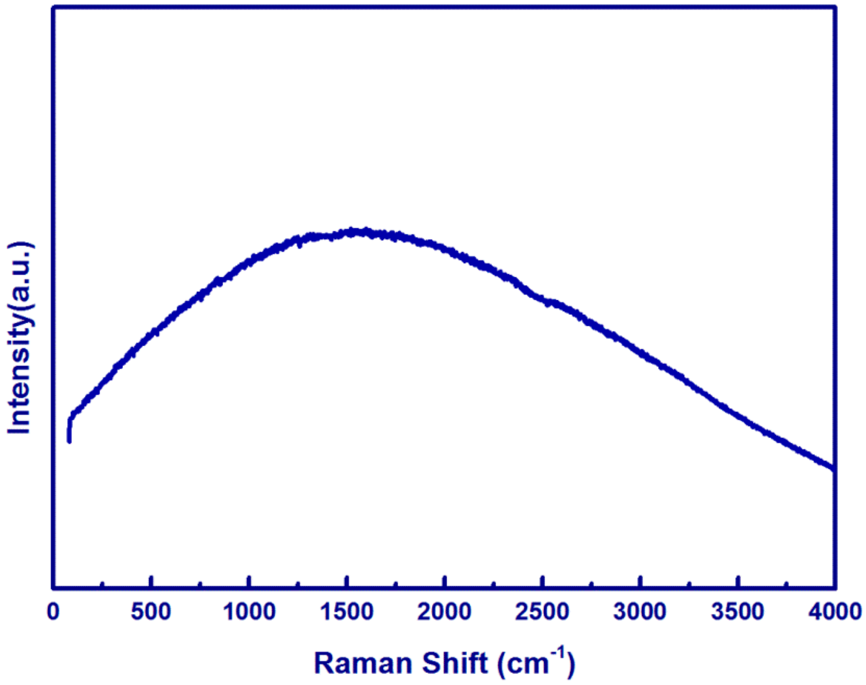

Figure S3. SERS spectrum of p-ATP (1×10^{-4} M) recorded on randomly selected spots on the surface of PAN/Ag.

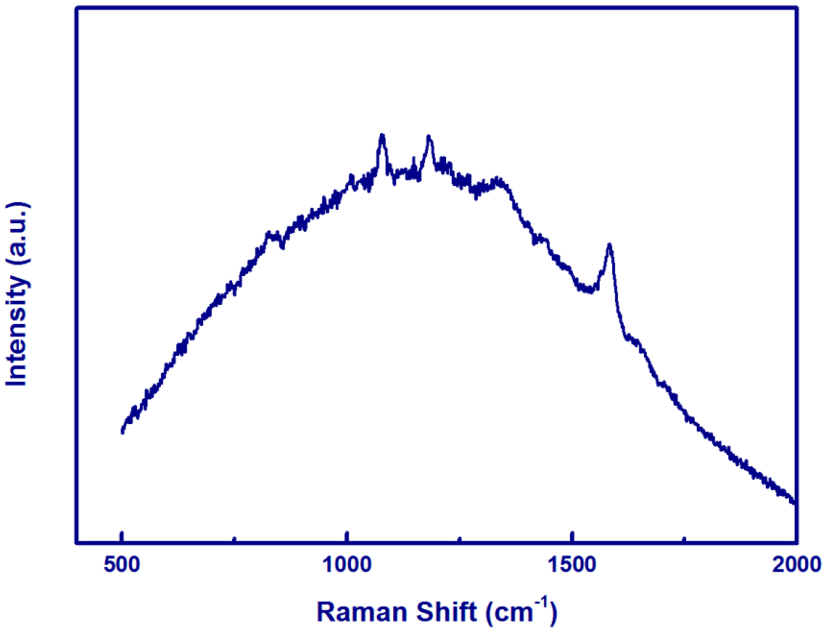

Figure S4. SERS spectrum of p-ATP (1×10^{-4} M) recorded on randomly selected spots on the surface of PAN/CNC/Ag.

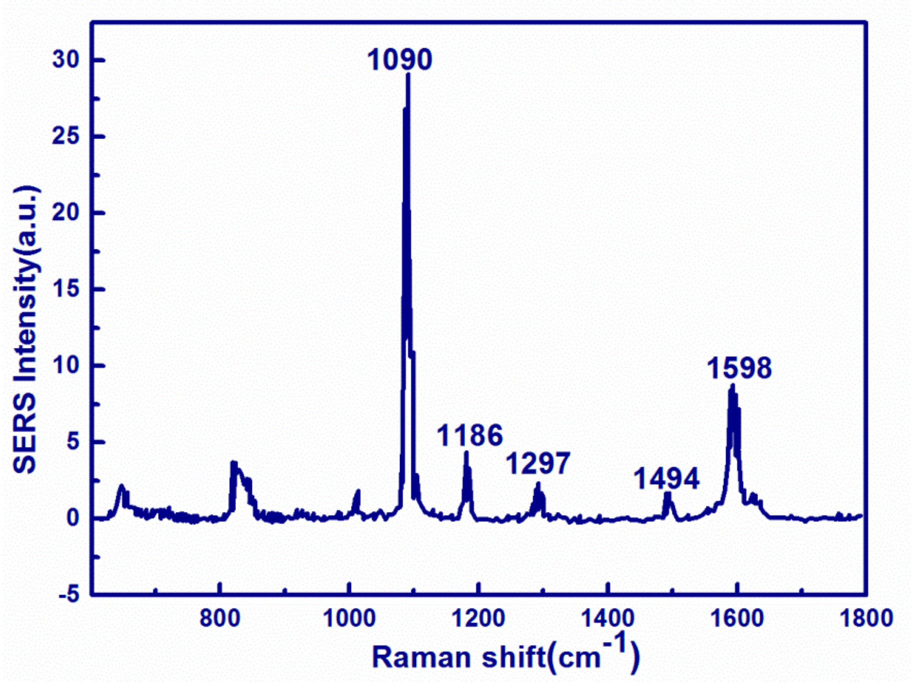

Figure S5. Normal Raman spectrum of solid p-ATP.
